# Iridovirus and Microsporidian Linked to Honey Bee Colony Decline

**DOI:** 10.1371/journal.pone.0013181

**Published:** 2010-10-06

**Authors:** Jerry J. Bromenshenk, Colin B. Henderson, Charles H. Wick, Michael F. Stanford, Alan W. Zulich, Rabih E. Jabbour, Samir V. Deshpande, Patrick E. McCubbin, Robert A. Seccomb, Phillip M. Welch, Trevor Williams, David R. Firth, Evan Skowronski, Margaret M. Lehmann, Shan L. Bilimoria, Joanna Gress, Kevin W. Wanner, Robert A. Cramer

**Affiliations:** 1 Division of Biological Sciences, The University of Montana, Missoula, Montana, United States of America; 2 College of Technology, The University of Montana, Missoula, Montana, United States of America; 3 US Army Edgewood Chemical Biological Center, Aberdeen Proving Ground, Edgewood Area, Maryland, United States of America; 4 Science Applications International Corporation, Abingdon, Maryland, United States of America; 5 Science Technology Corporation, Edgewood, Maryland, United States of America; 6 OptiMetrics, Inc., Abingdon, Maryland, United States of America; 7 Bee Alert Technology, Inc., Missoula, Montana, United States of America; 8 Instituto de Ecologia AC, Xalapa, Veracruz, Mexico; 9 Department of Information Systems and Technology, The University of Montana, Missoula, Montana, United States of America; 10 Department of Veterinary Molecular Biology, Montana State University, Bozeman, Montana, United States of America; 11 Department of Biological Sciences, Texas Tech University, Lubbock, Texas, United States of America; 12 Department of Plant Sciences and Plant Pathology, Montana State University, Bozeman, Montana, United States of America; 13 Department of Computer and Information Sciences, Towson University, Towson, Maryland, United States of America; 14 Center for Biotechnology and Genomics, Texas Tech University, Lubbock, Texas, United States of America; University of California Davis, United States of America

## Abstract

**Background:**

In 2010 Colony Collapse Disorder (CCD), again devastated honey bee colonies in the USA, indicating that the problem is neither diminishing nor has it been resolved. Many CCD investigations, using sensitive genome-based methods, have found small RNA bee viruses and the microsporidia, *Nosema apis* and *N. ceranae* in healthy and collapsing colonies alike with no single pathogen firmly linked to honey bee losses.

**Methodology/Principal Findings:**

We used Mass spectrometry-based proteomics (MSP) to identify and quantify thousands of proteins from healthy and collapsing bee colonies. MSP revealed two unreported RNA viruses in North American honey bees, Varroa destructor-1 virus and Kakugo virus, and identified an invertebrate iridescent virus (IIV) (*Iridoviridae*) associated with CCD colonies. Prevalence of IIV significantly discriminated among strong, failing, and collapsed colonies. In addition, bees in failing colonies contained not only IIV, but also *Nosema*. Co-occurrence of these microbes consistently marked CCD in (1) bees from commercial apiaries sampled across the U.S. in 2006–2007, (2) bees sequentially sampled as the disorder progressed in an observation hive colony in 2008, and (3) bees from a recurrence of CCD in Florida in 2009. The pathogen pairing was not observed in samples from colonies with no history of CCD, namely bees from Australia and a large, non-migratory beekeeping business in Montana. Laboratory cage trials with a strain of IIV type 6 and *Nosema ceranae* confirmed that co-infection with these two pathogens was more lethal to bees than either pathogen alone.

**Conclusions/Significance:**

These findings implicate co-infection by IIV and *Nosema* with honey bee colony decline, giving credence to older research pointing to IIV, interacting with *Nosema* and mites, as probable cause of bee losses in the USA, Europe, and Asia. We next need to characterize the IIV and *Nosema* that we detected and develop management practices to reduce honey bee losses.

## Introduction

### Honey Bee Colony Health in the USA

Colony Collapse Disorder (CCD) continues to impact bee colonies in the USA in 2010 at levels seemingly equal to, or exceeding that of 2007, when this unusual syndrome first received worldwide press coverage [1], [2]. The disorder is characterized by sudden losses of bees. This results in nearly empty beehives that, at best, may harbor a queen and a small worker bee population. A vexing aspect of the disorder is that there are ample resources left in the hive, and few or no dead bees in or near the hive. Bees seem to disappear without a trace [2], [3].

An unabated reappearance of CCD year on year demonstrates lack of progress toward solving the problem. Metagenomics initially identified Israeli acute paralysis virus (IAPV) as a potential marker or cause of CCD [4], yet a subsequent study demonstrated that IAPV was in the USA long before the recent CCD outbreaks [5]. While IAPV can affect honey bee health, the role of IAPV in CCD remains inconclusive. Proteomics analysis of other samples of bees from CCD colonies, conducted by the U.S. Army, yielded one of the earliest reports of *Nosema ceranae* and of Varroa destructor virus-1 (VDV-1) [6], as well as an RNA virus that was later identified as IAPV [7], in North American bees. Contrary to earlier indications, Army results indicated that IAPV was not present in all CCD-affected samples. RNA treatments have recently been reported to control IAPV and implied control of CCD [8], but it is not known whether IAPV causes CCD. A recent study failed to confirm a link between CCD and IAPV [9], and while IAPV can contribute to honey bee mortality, the signs are not consistent with CCD [10].

Other studies pointed to a variety of additional possible markers of CCD. A transcriptome study used gene expression-based techniques and identified an abundance and variety of RNA fragments in the gut of bees from CCD colonies. The authors suggested that these fragments were possible markers of CCD. Whether these RNA fragments were from the host or RNA bee viruses is unknown [11]. A survey of bee samples from across the USA revealed traces of pesticides in many bee samples, but none were shown to correlate with CCD [12]. Klee *et al.* [13] detected the microsporidian *Nosema ceranae* in bees from many countries, and scientists in Spain concluded that *N. ceranae* causes CCD [14]. USA researchers, on the other hand, have concluded that *N. ceranae* does not contribute significantly to CCD [3], [9]. *N. ceranae* has often been found in both healthy and failing colonies and its role in CCD in the USA remains unclear.

Given the diversity of potential microbes found in CCD colonies to date, and acknowledged environmental stresses faced by honey bees, some investigators have concluded that CCD is not a specific disease. It is rather a characteristic of colonies collapsing from an assortment of pathogens, physiological stress, or intoxications [9]. Still, this hypothesis fails to explain how or why these factors suddenly produce such a prevalent, highly distinctive, and unusual disorder. In addition, and perhaps importantly, whereas CCD has a precise set of signs in the USA [2], [3], it is not clear whether these are the same signs or causes of bee losses observed in other countries [15].

In this study, we used mass spectrometry-based proteomics (MSP) and a rigorous sampling method in an attempt to identify potential markers of CCD. MSP offered an orthogonal and complementary approach [16], [17] to gene-based techniques used in previous CCD studies for pathogen screening and classification. Mass spectrometry yielded unambiguous peptide fragment data that was processed by bioinformatics tools against the full library of peptide sequences based on both genomic and proteomic research. Consequently peptide fragment data acquired by MSP allowed identification and classification of microorganisms from the environment that was unrestricted by the need for amplification, probes, or primers. Furthermore, this approach allowed for the detection, quantification, and classification of fungi, bacteria, and viruses in a single analytical pass [18]–[19]. Classification can be to strain level and is limited only by the level of precision within the proteomic and genomic databases.

Our MSP analyses revealed the presence of two RNA viruses not previously reported in North American bee populations, as well as a highly significant and also unreported co-occurrence of strains of DNA invertebrate iridescent viruses (IIV) with a microsporidian of the genus *Nosema* in CCD colonies. The two RNA viruses were only seen occasionally, but the finding of the DNA virus in virtually all CCD samples establishes a new avenue for CCD research, as nearly all previous viral work to date in honey bees has focused on RNA viruses.

## Results

MSP analysis was used to survey microbes in bee samples from: (1) CCD colonies from the original event in 2006–2007 from widespread locations in eastern and western parts of the USA (2006–2007 CCD Colonies), (2) a collapsing colony in an observation hive fitted with a bi-directional flight counter and sampled through time as it failed in 2008 (2008 Observation Colony), (3) an independent collapse of bee colonies from CCD in Florida in 2009 (2009 Florida CCD), (4) packages of Australian honey bees delivered to the USA (2007 Australian Reference Group), (5) an isolated non-migratory beekeeping operation in Montana with no history of CCD (Montana Reference Group), and (6) dead bees recovered from inoculation feeding trials with *N. ceranae* alone, IIV alone, a mixture comprising *N. ceranae* plus IIV, and controls that were fed syrup alone in 2009–2010 (Inoculations Recovery Group).

MSP analysis resulted in a database of more than 3,000 identifiable peptides, representing more than 900 different species of invertebrate-associated microbes. An extensive summary of detected peptides and microbes is presented in a recently completed technical report [20]. We narrowed the list of suspect microbes to those infecting bees and insects, 121 in all. Of these, only 29 were specific to bees or occurred in more than one percent of the colonies sampled. These formed the subset of pathogens that we used for subsequent analyses. We focused our search on viruses, fungi, and microsporidia in the genus *Nosema*. We did not include well known bacterial infections of honey bees that are easily recognized, with visible signs that differ from CCD. We also observed *Varroa* mites in some, but not all of the CCD colonies.

Peptides were identified from nine of the approximately 20 known honey bee viruses in the initial sample set (Table 1). The isolated, non-migratory, Montana colonies that we included as a reference group were unique in that they were nearly virus-free except for a single colony that was positive for a low level of SacBrood virus (SBV) infection.

**Table 1:** Frequency (Frq) of occurrence and mean peptide counts of viral pathogens and *Nosema* in honey bee colonies sampled in 2006, 2007, and 2008^a^.

	East Coast – West Coast Colonies, 2006						Observation Colony, 2007		Florida Colonies, 2008	
	Collapsed n = 8		Failing n = 10		Strong n = 13		Subsamples = 18		n = 9	
Pathogen	Frq	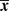 (s.d.)	Frq	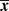 (s.d.)	Frq	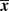 (s.d.)	Frq	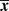 (s.d.)	Frq	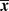 (s.d.)
**Acute bee paralysis virus**	2	0.3 (0.46)	5	1.5 (2.07)	5	0.9 (1.28)	13	1.3 (1.28)	7	11.6 (12.4)
**Black queen cell virus**	2	0.4 (0.74)	6	1.4 (1.8)	3	0.8 (1.54)	4	0.3 (0.57)	7	1.9 (1.5)
**Deformed wing virus**	3	0.8 (1.4)	1	0.2 (0.6)	6	0.6 (0.8)	4	0.6 (1.38)	7	15.9 (20.1)
**Iridescent virus**	8	20.9 (28.2)	10	38.0 (39.6)	9	15.6 (22.4)	18	16.1 (12.74)	9	57.6 (23.6)
**Israeli acute paralysis virus**	1	0.3 (0.7)	4	1.4 (2.3)	5	0.8 (1.3)	11	0.9 (0.96)	5	2.4 (2.8)
**Kakugo virus**	0	0 (0)	0	0 (0)	3	0.3 (.08)	3	0.2 (0.55)	2	0.3 (.04)
**Kashmir bee virus**	3	0.2 (3.2)	6	1.9 (2.1)	9	1.0 (0.9)	1	1.0 (1.28)	6	3.6 (5.0)
**Sacbrood virus**	2	0.9 (1.6)	4	0.9 (1.4)	6	1.2 (2.3)	11	1.3 (1.36)	6	3.8 (7.0)
**Varroa destructor virus 1**	0	0 (0)	1	0.2 (0.6)	1	0.2 (0.6)	4	0.4 (1.04)	5	1.3 (1.6)
***Nosema*** ** group 1**	5	6.4 (9.1)	9	11.4 (9.6)	7	5.2 (7.7)	18	8.7 (5.74)	9	35.2 (15.3)
***Nosema*** ** group 2**	3	0.8 (1.4)	3	0.7 (1.3)	3	0.2 (0.4)	11	1.0 (0.97)	0	0 (0)

a Columns summarize thirty-one colonies from initial CCD study in 2006; eighteen subsamples taken from an observation colony monitored through its collapse from March through August 2007; and a third sample of nine colonies sampled during a CCD incident in Florida in 2008. A hyphen indicates that the value could not be calculated.

The recently-described Varroa destructor virus 1 (VDV-1) [21] was detected in two colonies from the 2006–2007 CCD colonies; one from an eastern, and one from a western location [6], [20]. Peptides of Kakugo virus [22], [23], which previously has not been reported in North American bees, were detected in two colonies from a single west-coast location in this same CCD group of colonies.

IAPV did not occur frequently and was distributed approximately equally among strong and failing colonies (Table 1). It was more prevalent in colonies that originated from the East Coast of the USA (four of ten) and Australia (three of ten).

The most prevalent viral peptides we detected were identified as invertebrate iridescent viruses (IIV), large double-stranded DNA viruses of the *Iridoviridae* family. We detected 139 unique peptides in west- and east-coast colonies that were attributed only to IIV type 6 (IIV-6, also known as Chilo iridescent virus) with high confidence (≥0.99).

IIV appeared with 100 percent frequency and at higher peptide counts in failing and collapsed colonies. IIV also occurred in nearly 75 percent of strong colonies, although invariably at lower concentrations. Numerous peptides for *Nosema* species were detected in collapsed and failing colonies. Peptides attributed to ten species of *Nosema* were represented, but because of high cross-correlations among the different peptides within the genus these were aggregated based on cluster analysis into two distinct groupings.

Using those groupings we observed that one group of *Nosema* peptides paralleled the pattern of occurrence for IIV (*r* = 0.90, n = 31, *P*<0.001) and was present at higher frequency more often in failing and collapsed colonies than in strong colonies (Table 1). Further suggestive correlations with other microbes included the co-occurrence of Black queen cell virus (BQCV) and IIV (*r* = 0.71, n = 31, *P*<0.001) and concordantly the same *Nosema* group (*r* = 0.73, n = 31, *P*<0.001).

Count-weighted occurrence data were subjected to stepwise discriminate function analysis to assess whether strong, failing, or collapsed colonies could be differentiated by specific patterns of pathogen occurrence. The isolated Montana apiary was included as a non-CCD reference group for this analysis. The colonies in this group served as an external control group that was complementary to the strong colonies within the CCD apiaries that served as internal controls.

**Figure 1:**
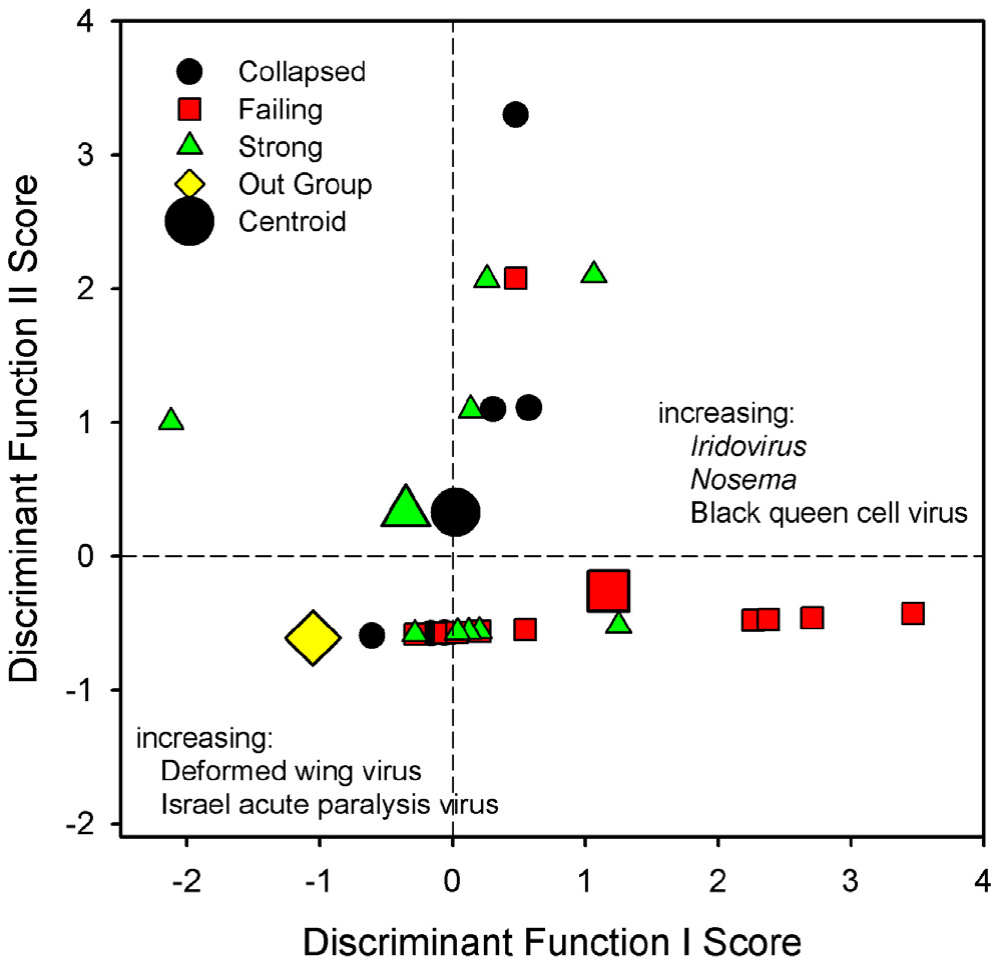
Discriminant Function Analysis for differences in pathogen peptide counts among strong, failing, and collapsed honey bee colonies. Function 1 explains 81 percent of discriminating variance and contrasts higher incidence of iridovirus (IIV), *Nosema*, and to a lesser extent BQCV in failing colonies with higher incidence of DWV and some IAPV in the remaining groups. Vertical and horizontal lines mark the non-CCD out-group as a reference set.

Discriminate analysis indicated that only two pathogens, IIV and Deformed wing virus (DWV) were necessary for significant discrimination among different colony groups (Table 2). The first function contrasted higher incidence of IIV in failing colonies with higher incidence of DWV in the remaining groups (Figure 1). The structure matrix (correlations with discriminant functions) showed that *Nosema* 1 was the highest correlated variable among those not included with the discriminant functions (Table 3, *r* = 0.69). This indicates that the incidence of IIV and *Nosema* 1 were strongly associated with group scores on the discriminant functions.

**Table 2:** Cumulative variance, significance, and coefficients for derived discriminant functions.

								Standardized Function Coefficients	
Function	Eigen value	Var. %	Cum. %	Canonical Correlation	Chi-square	df	*P*	IIV-6	DFW
**1**	0.68	80.6	80.6	0.64	22.8	6	0.001	1.17	−0.65
**2**	0.16	19.4	100.0	0.38	5.2	2	0.076	0.05	0.98

**Table 3:** Pooled within-groups correlations between discriminating variables and standardized canonical discriminant functions ordered by absolute size of correlation within function.

Structure Matrix		
	Function	
Pathogen	1	2
Invertebrate iridescent virus 6	0.83	0.55
*Nosema* group 1	0.68	0.60
*Nosema* group 2	0.60	0.34
Black queen cell virus	0.59	0.53
Acute bee paralysis virus	0.51	0.09
Israeli acute paralysis virus	−0.13	−0.02
Deformed wing virus	−0.04	0.99
Sac brood virus	0.15	0.60
Kashmir bee virus	0.40	0.49

As expected, the Montana reference group was most distinct and significantly different from the strong condition colonies (*P*_out – strong_ = 0.06, *F* = 5.5, df = 2,33; *P*_out – failing_<0.001, *F* = 17.3, df = 2,33; *P*_out – collapsed_ = 0.04, *F* = 7.5, df = 2,33). Failing colonies were distinct from both good and reference colonies (*P*_failing-strong_ = 0.002, *F* = 7.4, df = 2,33; *P*_failing-out_ = 0.001, *F* = 10.1, df = 2,33) based mostly on differences in IIV peptide abundance. The only anomaly was that collapsed and strong colonies were not significantly different (*P*_collapse-strong_ = 0.71, *F* = 0.3, df = 2,33). This similarity between collapsed and strong colonies seems contradictory at first.

It is, however, likely that the few bees left in colonies at the final stages of collapse are those that are not infected, and thus would be expected to be similar to uninfected bees in strong colonies. *Nosema* species by themselves were not a significant predictor of colony condition, but *Nosema* group 1 was highly correlated with IIV (*r* = 0.901, n = 31, *P*<0.001), and so was not selected by the stepwise procedure because of its statistical association with IIV.

**Figure 2:**
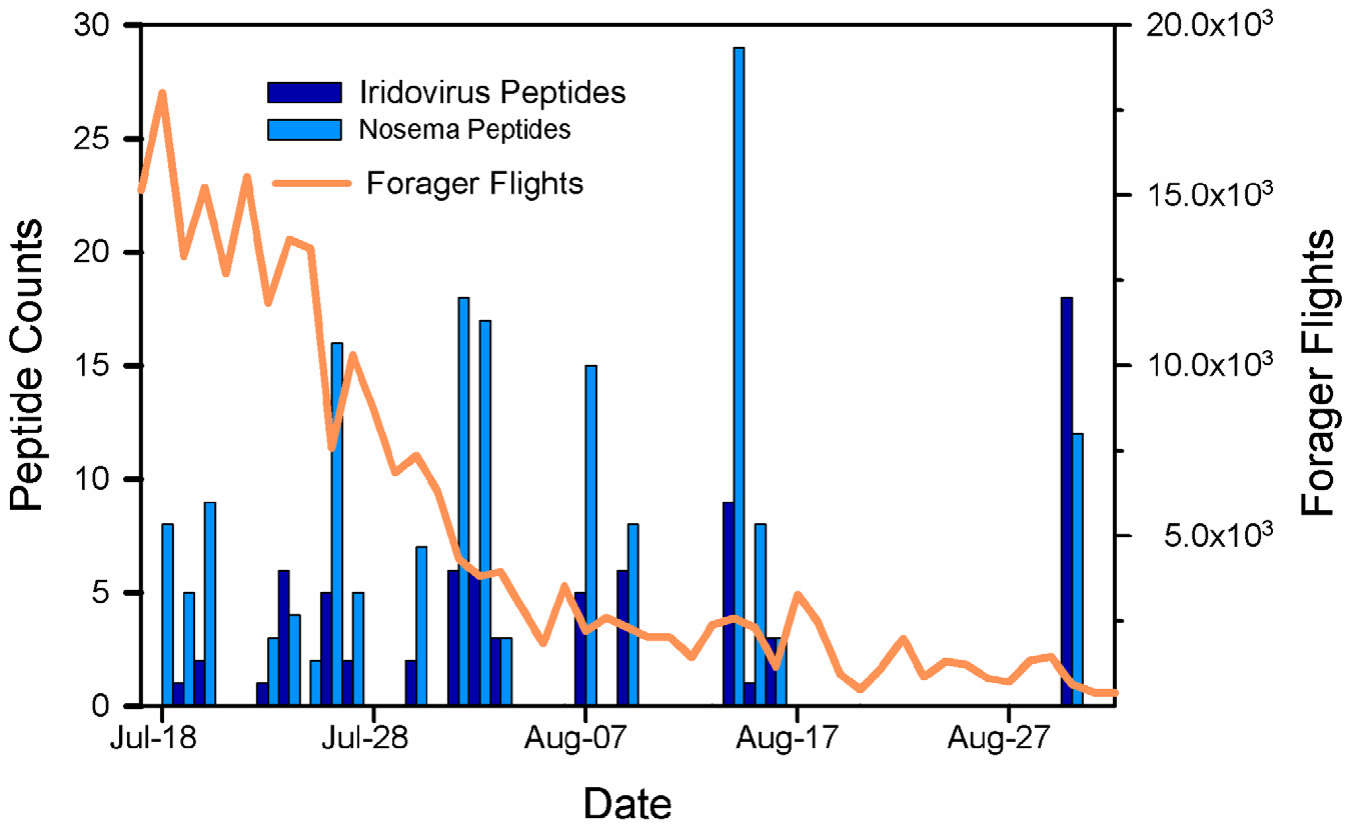
Decline in forager flights in conjunction with increasing counts of iridovirus (IIV) and *Nosema* peptides. The peptides were detected in dead worker honey bee samples collected from a single collapsing observation hive at the University of Montana, Missoula. Forager flights are absolute counts per day, tabulated by an automated honey bee counter. Peptide counts are the summed counts by day of collection for all unique IIV *Nosema* peptides in each sample. We suspended sampling in mid-August, as the bee population was too weak to sample without affecting the colony. We collected the queen and last few bees for a final sample in early September. Sample size varied from about ten bees to more than 100 per sample interval, depending on how many dead bees were obtained from the entrance tube.

In the 2008 observation colony, as CCD progressed, flight activity exhibited several peaks and crashes until it declined by approximate geometric decay to extinction (Figure 2). Only nine common RNA bee viruses were identified by proteomics, and most occurred in only one or a few samples, with little correlation to the progression of collapse (Table 3). IIV and *Nosema*, however, occurred throughout the collapse.

Both of these pathogens increased when the bee population decreased most sharply and remained at high levels throughout the remainder of the collapse. None of the other pathogens found in the colony showed a similar pattern. Polymerase chain reaction (PCR) analysis of the *Nosema* species present in this observation colony revealed the presence of *N. ceranae* alone (data not shown).

The 2009 Florida CCD samples presented an independent opportunity to corroborate our findings. A group of nine colonies, in sets of three, identified as either strong, failing, or collapsed, were sampled and analyzed. We used the classification functions generated in our original discriminant function analysis from 2007 to classify these new samples as either strong, failing, collapsed, or out-group reference based on virus and *Nosema* patterns. Of the nine colonies, six were classified as either collapsed or failing. None were classified as the healthy reference group, leaving the remaining three classified as strong. The analytical classification was close to the original designation for these colonies. Two of the three originally identified strong colonies were classified as such; one was classified as failing. Differences between failing and collapsed designations were not as distinct, but were consistent in classifying five of six colonies as suffering from CCD based on IIV and *Nosema* occurrence.

We detected 139 unique peptides in our west and east-coast data that were attributed to IIV-6 with high confidence (match to index ≥0.99). Later samples also indicated an IIV-6-like virus as the dominant virus in collapsing colonies (88 percent of IIV peptides). Furthermore, comparison of IIV peptides among all samples revealed moderate but significant correspondence between the original samples, the laboratory inoculation experiments, and the other field samples (Table 4).

**Table 4:** Similarity in occurrence of specific iridescent virus peptides among different samples analyzed for evidence of pathogens associated with CCD.

Sample		Florida Collapse	Inoculation Trial	Collapsing Colony
**Inoculation Trial**	*Rho* a(*P*_two-tailed_)	0.26 (0.00)		
	Sorensen's Index^b^	0.18		
**Collapsing Colony**	*Rho* (*P*_two-tailed_)	0.08 (0.21)	0.11 (0.07)	
	Sorensen's Index	0.21	0.18	
**East-West CCD Colonies**	*Rho* (*P*_two-tailed_)	0.30 (0.00)	0.22 (0.00)	0.03 (0.66)
	Sorensen's Index	0.58	0.20	0.17

a Spearman's rank correlation (rho; n = 266).

b Sorensen's index of similarity were calculated for each pairwise comparison. East-, West-CCD colonies sampled 2007–2008; Collapsing Observation Colony, 2008; Florida Collapse, 2009; Inoculation Trials, 2009–2010.

These procedures may have identified IIV-6 as the most likely source of peptides because this is the only fully sequenced genome from the genus *Iridovirus*. We suspect that bees may in fact be infected by IIV-24 that is also assigned to the *Iridovirus* genus, which was isolated from an Asian bee [24]–[26], or by a variant of IIV-6. Isolation of the unknown IIV we detected is not a trivial matter. Virus isolation is usually achieved using in vitro cell culture techniques, but this is not easily achieved from CCD bees because many are infected by multiple viruses and plaque purification techniques are not available for most of these viruses. Nonetheless, attempts at viral isolation are ongoing.

Our original wide area bee samples from 2007, the time series samples from the collapsing observation colony from 2008, and the reoccurrence of CCD reflected by Florida samples from 2009 were consistent in that IIV-6 peptides plus some IIV-3 peptides were the only IIV entities detected and were correlated with infections by *Nosema* species. Isolation of the IIV(s) and *Nosema* species in our samples is not trivial and is the subject of our ongoing research. Isolates, however, of a strain of IIV-6 and *N. ceranae* were immediately available. Thus, to test our MSP generated hypothesis that an interaction between *N. ceranae* and IIV leads to increased bee mortality we conducted inoculation cage-trial experiments.

**Figure 3:**
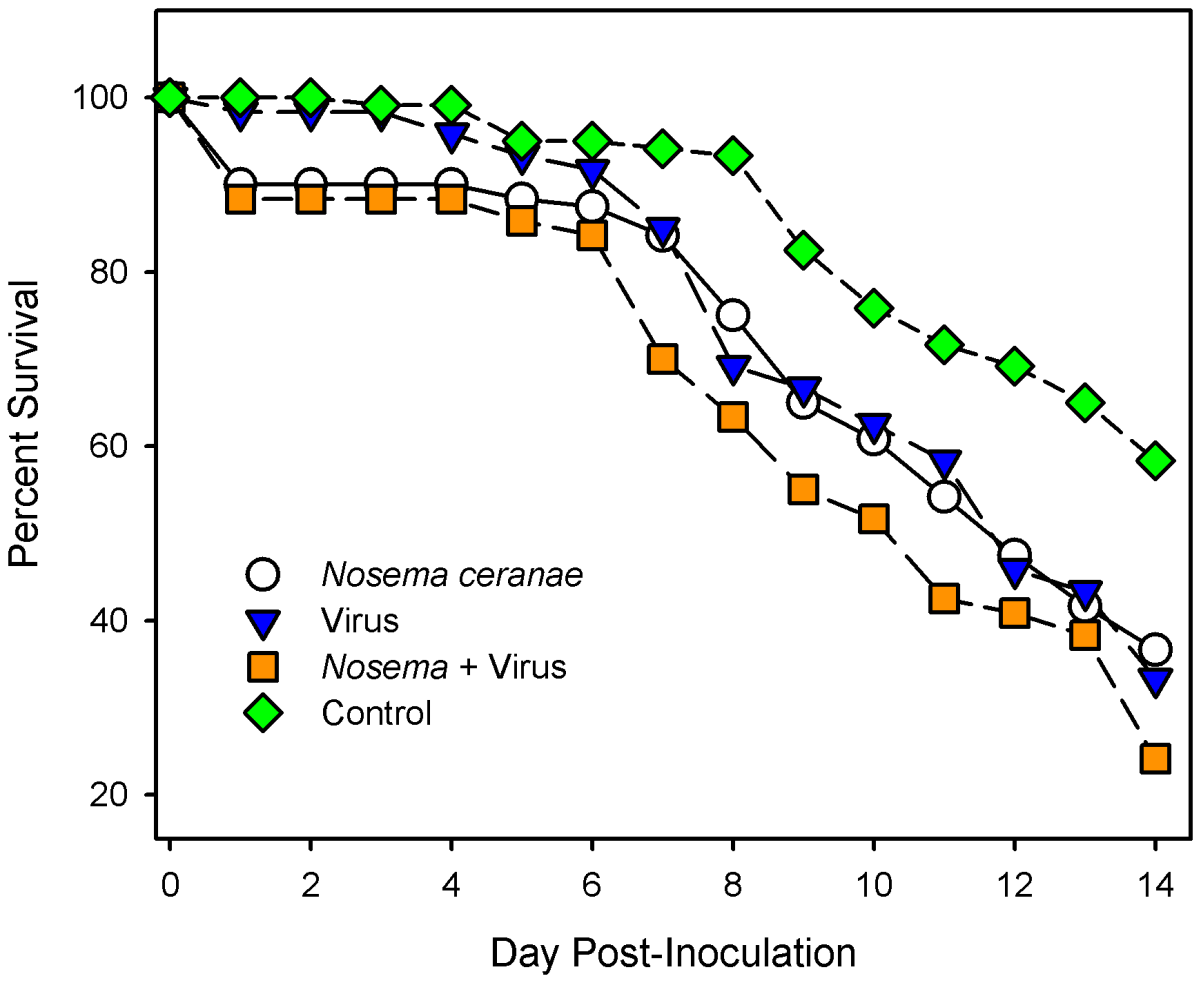
Survival over a fourteen day post-infection period observed in cage-trials of *Nosema ceranae* and IIV infected honey bees. Newly emerged, 1–3 day old, bees were used in all experiments. Figure represents the combined survival results for 4 biological replications (N = 30 bees in each group for each biological replicate). Bees that perished within 24 hours of inoculation were not included in the survival curve analyses. Deaths in control group were confirmed not to be pathogen related via mass spectroscopy analyses. Inoculum sizes and doses described in materials and methods section. Log Rank Tests Kaplan-Meier Curve Analyses: 1) Control VS. *N. ceranae* - P = 0.01, 2) Control VS. IIV alone – P<0.01 (0.008), 3) Control VS. *Nosema *+ IIV – P<0.01 (0.0001), 4) *Nosema* alone vs. IIV alone – P = 0.90, 5) *Nosema* alone VS. *Nosema *+ IIV – P = 0.04, 6) Virus alone VS. *Nosema *+ IIV – P = 0.04. These results strongly suggest that the combination of *N. ceranae* and IIV is associated with increased bee mortality.

Cage trials of 1–3 day old newly-emerged bees demonstrated increased mortality in the experimental group fed both *N. ceranae* and IIV-6 in comparison with the control group (P = 0.0001) and bees fed only *N. ceranae* (P = 0.04) or only IIV-6 (P = 0.04, Figure 3). As the actual infectious dose of *N. ceranae* or IIV that occurs in the field is currently unknown, we chose to utilize a relatively low infectious dose for both pathogens in our experiments. As is common in cage bee trials, mortality was observed in the control groups in all four biological replicates. To confirm that the controls likely died from a non-infectious cause, deceased bees from all treatment groups were further screened with MSP. The controls did not have any detectable IIVs, but did show some evidence of *Nosema*, which was not apparent from PCR analysis of the same samples.

These results revealed mostly an absence of pathogens in the control bees, and the presence of peptides related to IIV-6 or *N. ceranae* in the appropriate groups (data not shown). Importantly, no statistical difference in survival was observed between the bees fed *N. ceranae* or the virus alone. These results support the correlation observed by the MSP data that suggests than an interaction between *N. ceranae* and an IIV-6-like virus may be involved in bee mortality. Whether an additive or synergistic interaction would be observed between *N. ceranae* and other bee viruses is currently unknown, but merits further study.

## Discussion

MS-based proteomics provided an unrestricted and unbiased approach for surveying pathogens in honey bee colonies. Our results detected a DNA virus and two RNA viruses that had not been previously reported in honey bees from the USA. The potential correlation of IIV with CCD may previously have gone unnoticed because these are large DNA viruses, not the small RNA viruses commonly considered to be the cause of most bee diseases. The correlation between IIV and *N. ceranae* for bee colonies exhibiting CCD implies that they track each other.

Interestingly, the presence or absence of IIV in a given honey bee colony may explain why in the USA *N. ceranae* sometimes seems to contribute to severe colony losses (IIV present), and sometimes not (IIV absent), as reported both by researchers and beekeepers [1], [3], [4], [9]. The mechanism by which these two pathogens interact to potentially increase bee mortality is unknown. It may be that damage to gut epithelial and other host cells by the *N. ceranae* polar tube allows more robust entry of the virus. Alternatively, replication of *N. ceranae* in honey bee cells may cause a decrease in the bees' ability to ward off viral infections that normally could be controlled. These types of studies await isolation of the definitive virus.

Other than the presence of the IIV, only the concurrent absence of Deformed wing virus, another RNA virus, was significant with respect to CCD. We recognize that the iflaviruses VDV-1 and Kakugo virus appear to be variants of Deformed wing virus, whereas the dicistroviruses Kashmir bee virus, Acute bee paralysis virus and IAPV are also closely related to one another [27]. Even closely related viruses may express different etiologies, and as such, we treated them as separate variables in our statistical analyses.

Virtually all of the bees from CCD colonies contained *Nosema* species and IIV, whereas IIV was not found in bees from packages imported from Australia nor in bees from colonies of the non-migratory, commercial bee operation in Montana. CCD has not been reported by the Australians [28] or the Montana beekeeper.

Since inapparent non-lethal infections by IIVs are common [29], detection of IIV in some strong colonies and in the remnant young bee populations of collapsed colonies is to be expected. Presence of IIV in bees located in strong colonies may indicate a non-lethal infection, an early stage of the disease, or possible resistance to the virus in these colonies. In collapsed colonies, the presence or absence of IIV in remnant young bee populations is likely dependent upon the extent of infection in the colony and unknown pathophysiological factors that may affect persistence, colonization, and replication of the virus. Development of a PCR- or antibody- based assay to detect IIV in honey bees will allow us to rapidly detect and track the presence of IIV throughout the honey bee life cycle in a given colony.

Large amounts of IIV in failing colonies is consistent with an infection that proliferates in bees but not necessarily to a degree that results in the iridescent coloration of infected bee tissues that is characteristic of IIV disease. The sustained high levels of IIV and *Nosema* peptides that occurred in the observation colony as the frequency of forager flights declined strongly implicates an IIV-6-like virus and *Nosema* as co-factors in CCD, since forager bee activity declined as pathogen loads peaked. In Spain, researchers have published studies linking *N. ceranae* to colony collapses in that country, yet we noticed that pest and disease surveys have reported the presence of an iridescent virus in colonies surveyed for mites and diseases [30] .

Because of their virulence, IIVs have been investigated as candidates for use as biopesticides [31]–[33]. Invertebrate iridescent viruses (IIVs) are large, icosahedra, double-stranded DNA viruses [29], [34]. Of the many isolates reported from insects, only two, IIV-3 and IIV-6 [29], [35], [36] have been subjected to complete genome sequencing and an additional 24 have been partially characterized [29], [37], [38]. Historically, IIVs were numbered according to date of isolation [37], [38]. Uniformly packed particle arrays [34], [40] of these viruses produce opalescent colors in the tissues of heavily infected hosts, particularly in insects in damp or aquatic habitats. These viruses have been shown to alter insect growth, longevity, and reproduction, and induce cell apoptosis [29], [34], [39], [40]–[46]. In silkworms, IIV-1 can induce epidermal tumors [47].

Patent IIV infections are almost invariably lethal but inapparent or covert infections may be common [29], [34]. Inapparent infections may not be lethal, but may affect the reproduction and longevity of covertly infected hosts [42]. IIV-3 is thought to be restricted to a single species of mosquito [34], [36], although we found peptides close to those of IIV-3 in bees from an observation hive. These bee samples were hand-picked with forceps, so we are confident that our observation hive samples did not include mosquitoes or other insects.

Other IIVs, such as IIV-6, naturally infect various species of Lepidoptera and commercial colonies of Orthoptera. There is evidence that hymenopteran endoparasitoids can become infected if they develop in an infected caterpillar [45]. IIVs have also been studied for control of mosquitoes [32], [33] and boll weevil [31], the latter work examined the virus itself, with an emphasis on the proteins that it produces as the basis for a possible biopesticide. A U.S. Patent has already been awarded [31].

There is one known iridescent virus in bees. IIV-24, originally isolated from the Asiatic honey bee *Apis cerana*, severely affects bee colonies, causing inactivity, crawling, and clustering disease [24]–[26]. Proteomics could not identify IIV-24 in any of our samples because there are no IIV-24 sequences in the current databases. Thus, thus the identity of the IIV in our samples remains undetermined.

Based on the sequence data generated from MSP, the IIV identified appears to be closely related to IIV-6, possibly because this is the only IIV in the *Iridovirus* genus that has been completely sequenced. The major capsid protein represents approximately 40 percent of the total particle polypeptide and is highly conserved, so sequencing peptide fragments may frequently identify IIV-6 as being the most likely candidate [37], [40]. This argument is reinforced by some results coming back as IIV-3, which is presently assigned to a different genus in the family (*Chloriridovirus*) and only reported to occur in a mosquito [36].

There is little information about IIVs in bees, although there are historical reports associating IIVs with severe bee losses in India [24]–[26], the U.S. [48], and possibly Spain [30]. In the 1970s, in northern India, almost every bee was infected with IIV-24, with 25–40 percent annual colony loss [25], [26]. The disease was manifested by inactivity, clustering, and crawling sickness.

Transmission of IIV-24 is suspected to occur via eggs, feces, or glandular secretions in food [25], [26]. Evidence that IIV-24 was the cause of Indian bee losses was based on turquoise and blue iridescence seen in affected bees and tissues, serological tests, and microscopic examination of sick bees. IIV was the only recognizable parasite in all samples. IIV-24 was strongly correlated with co-infective *Nosema* species and tracheal mites in diseased colonies of *Apis cerana* [25], [26]. Tracheal mites were found in some, but not all of the sick colonies [24], [26]. The fat body was always attacked by the virus, and other tissues and organs, including the ovaries were frequently infected [24], [26].

In addition, an iridescent virus has also been associated with mites, which may act as vectors, and has been implicated in bee losses in the United States. While investigating unusually high losses of bees in the northeastern United States, Camazine and Liu [48] extracted a putative iridovirus from *Varroa* mites collected from a colony that perished four weeks later. They concluded that viral transmission within the colony might kill both mites and bees, but they were not able to discover the virus in time to determine whether bees in the colony were infected, and they were unable to purify the virus or determine whether the virus could be transmitted to bees by inoculation.

One or more species of external mites were suspected of being carriers of the virus in Indian bees [24]–[26], as was also the case in the U.S., with *Varroa* acting as the vector [48]. The need for a better knowledge of the ecology of IIVs has been emphasized in order that preventive measures could be taken to not only offset damage to *Apis cerana* but also to reduce the chance that *Apis mellifera* could become infected by this pathogen [26]. Indeed, IIV-24 was experimentally inoculated and found to lethally infect *A. mellifera*, forming cytoplasmic quasi-crystalline aggregates of virus particles in cells of the fat body, hypopharyngeal glands, the gut wall, and proximal ends of the Malpighian tubules [24], [26].

These historical findings of IIV, mites, and *Nosema* species are intriguing since researchers studying both *Nosema ceranae* and CCD in Spain observed IIV-like particles in bee samples by electron microscopy [30]. U.S. investigators studying CCD observed structures in thoraxes of bees described as ‘peculiar white nodules’, resembling tumors, that contained crystalline arrays [3], similar to those described for IIV infections. Also, it appears that the IIV-6 genome encodes for one or more polypeptides that can produce insect mortality by inducing apoptosis without the need for viral replication [43].

The suspected source of *Nosema ceranae* in *Apis mellifera* is the Asian bee *Apis cerana* [49]. This bee species is also known to be infected by Thai sacbrood virus and by Kashmir bee virus. Kashmir virus was first detected as a contaminant in a sample of iridescent virus from India, as well as IIV-24 [50]. The same virus was linked to bee losses in Canada in the early 1990s [51]. This suggests that perhaps not only the microsporidium *N. ceranae*, but other pathogens as well may have jumped from *Apis cerana* to *Apis mellifera*, as predicted by Bailey and Ball in 1978 [26].

It also implies that if Kashmir bee virus has been in North America for more than twenty years, so might IIV and *Nosema ceranae*. That would fit the time line of the first observations of this complex of pathogens, and of severe bee losses in India in the 1970s. It also leads us to ask whether the first widespread losses of bees in the USA, described as Disappearing Disease in the 1970s [52], may have been early outbreaks of CCD.

Our own work, described here, provides multiple lines of correlative evidence from MSP analysis that associate IIVs and *Nosema* with CCD in the USA. We conclude with results of laboratory inoculations of caged bees with IIV and *Nosema* that demonstrate the potential for increased lethality of mixed infections of these two pathogens. Our study strongly suggests a correlation between an iridescent virus, *Nosema*, and CCD. Our inoculation experiments confirmed greater lethality of an IIV/*Nosema* co-infection compared to infections involving each pathogen alone. Future research using the specific strains of IIV isolated from infected bees will surely confirm whether a synergistic or additive interaction between these two pathogens results in the signs and symptoms of CCD.

The fact that IIV-6 inoculated bees experienced increased mortality in the presence of *Nosema* clearly strengthens the significance of all lines of evidence pointing to an interaction between an IIV and *Nosema ceranae*. Lack of a stronger effect by preparations containing IIV-6 may be due to the possibility that the IIV detected by proteomics is either a strain of IIV-24 or a strain of IIV-6 that is more specifically adapted to honey bees, and consequently more virulent than the strain of lepidopteran origin used in our inoculation experiments. It is, of course, critical to isolate the IIV from CCD populations, compare it to known IIVs and particularly IIV-24, and then challenge CCD populations with this strain. This work is in progress.

Moreover, we used a fairly low dose of IIV-6 and *Nosema ceranae* spores. For example, IIVs are generally not highly infectious by ingestion. Similarly, virulence studies on *N. ceranae* have reported using over 200,000 spores per bee in cage trials whereas we used a four-fold lower dose. It will also be interesting to test whether the interaction between IIV and *N. ceranae* is specific, or a general “stress” phenomenon that could also be reproduced by addition of *N. ceranae* and any additional bee virus.

In our studies, we applied six independent scenarios to the assessment of potential causes or markers of CCD and got the same answer, giving us confidence in the results, since this inference approach is approximately analogous to applying the same technique to six different assessments [53]. Our results also provide credibility to older, often overlooked work by others that associated IIV with bees, tracheal and *Varroa* mites, *Nosema* species and severe bee losses. In our samples, *Varroa* mites were seen in many CCD colonies, but not in all. Importantly, our limited results do not completely fulfill the requirements of risk characterization, nor do they clearly define whether the occurrence of IIV and *N. ceranae* in CCD colonies is a marker, a cause, or a consequence of CCD. Our findings do make a strong case for a link between an Iridescent virus and *Nosema* with CCD and provide a clear direction for additional research to answer these questions.

We anticipate that there also may be questions as to why IIV was detected in our study, but has not been found in any current published research on CCD. And, if these viruses were present, why weren't they seen in infected tissues of the European honey bee, *Apis mellifera*?

First, iridescent viruses have been seen before in *Apis mellifera*, both in Europe and in the USA. Researchers in Spain reported seeing iridescent virus in honey bees [30], and Camazine [48] saw a putative iridescent virus in *Varroa* mites following a collapse of colonies in the northeastern part of the U.S. in the 1990s. Also, inapparent infections by iridescent viruses may involve a low density of IIV particles in infected host cells [46], so without sensitive techniques such as MSP, it is not surprising that infections in CCD bee colonies were previously missed.

The large number of IIV proteins that we identified, 139 in all, represent a significant fraction of the total IIV proteome. The recently published genome for IIV-6 [54] suggests a total proteome of 137 unique proteins. The 139 polypeptides identified for the IIV strain in our study must therefore represent a near complete sample of the total viral proteome belying any criticism that our identification of IIV may be a spurious consequence of accidental matching of a few peptide fragments.

We conclude that the IIV/*Nosema* association may be critical in honey bee mortality linked to CCD. Although viral diseases are currently manageable only by culling, *Nosema* infections are treatable with several current management techniques. We suggest that for beekeepers suffering from colony losses, disruption of the potential IIV/*Nosema* relationship using treatments that are available to control *Nosema* species may be one option to help reduce honey bee mortality. Again, whether this identified bee IIV and its potential interaction with *Nosema* species is the cause or marker of CCD, is unknown, but our results clearly suggest that further research in this area is urgently required.

## Methods

### Wide Area Bee Sampling

We collected sample sets of adult worker honey bees from several areas and years: (1–2) Two initial sample sets of adult honey bees from CCD colonies were obtained in 2006–2007 from twelve beekeeping operations from western, northeastern, and southeastern regions of the USA, (3) Samples from packages of imported Australian bees provided a non-CCD 2007 reference, (4) bees sampled in 2008 from a large, non-migratory beekeeping operation in northwestern Montana with no history of CCD provided a second reference set, (5) bee samples obtained in 2009 from a Florida apiary when 500 colonies suddenly collapsed constituted an independent CCD sample set by location and year.

In each apiary investigated and sampled for this study, based on visible signs of CCD as described by the CCD Working Group [3], samples of 200–500 bees were collected from each of nine colonies judged to represent the three most populous, three failing, and three collapsed colonies. Our team was part of the CCD Working Group that investigated and sampled the first reported colonies with CCD in the USA [3]. We later published an expanded description of the signs of CCD and variations that occur with season and geographical area, and we have continued to inspect and sample colonies showing signs of CCD from many areas of the U.S. from December of 2006 to the present. As such, we are well familiar with the signs and stages of CCD.

Typically, the largest colony populations had 10–14 frames of adult bees or more, and two or more frames of brood. The collapsed colonies had less than a frame of bees, often no more than the queen and a fist-sized cluster of very young bees. Failing colonies were defined as those that had no more than half the number of bees as the most populous colonies. These colonies often had an excess of bees, and had had far more bees just days or a few weeks before the samples were taken, according to the beekeeper accounts.

Additional reference bees were obtained from packages shipped from Australia to the USA and from the most populous to the weakest colonies from apiaries of a large, commercial beekeeping operation in Montana that is geographically isolated and has no history of CCD. In this case, the weakest bee populations were only about 20 percent smaller than the largest bee populations.

All of the CCD operations were large, migratory beekeeping businesses that transported bees across state borders and rented colonies for pollination of almonds in California. The migratory colonies sampled in 2006 and 2007 represented two different migratory routes, one from the east coast to California, the other from North Dakota to California. In addition, when in California, the east coast and the mid-western colonies were separated by approximately 400 kilometers, so that there was no overlap of either the apiary locations or highways of these two different migratory routes.

Bees were shaken directly into new, clean one quart Ziploc® or one liter Whirl-Pac ® bags. The bags were sealed, placed in a cooler with frozen gel packs, and shipped by overnight express to the U.S. Army Edgewood Chemical and Biological Center (ECBC) laboratory. Bees were often alive when received and were analyzed immediately. In a few cases, bee samples were frozen and stored in a −80°C freezer until analyzed.

Following the same sampling methods, we sampled a repeat of CCD in Florida, where 500 honey bee colonies started from packages in October of 2008, collapsed in January of 2009. As mentioned before, the beekeeper who owned the colonies had experienced CCD in 2006–2007, and had been one of the original beekeeping operations sampled by members of the CCD Working Group. As in 2006–2007, the colonies suddenly collapsed, demonstrating the characteristic signs of CCD [2], [3].

### Time Sequence Bee Sampling

We also observed the progression of CCD in a collapsing colony in an observation hive, taking 18 bee samples of approximately 10–60 bees per sample interval, over a three month period, ending when only a queen and four workers remained.

In the spring of 2008, we lost more than more than 50 of our research colonies to CCD. We took the frames, queen, and the small, surviving population of young bees from one of these collapsed colonies, put them in a five-frame observation hive, and fed them sucrose syrup. This colony soon produced a second queen, and both queens co-existed in the same colony, one on each side of the glass hive, together producing a rapidly increasing combined population of bees. The forager bees had access to both syrup and to abundant food resources from the University of Montana campus, UM's arboretum, and surrounding residential flower gardens. By mid-summer, this bee colony collapsed for the second time. We then began to sample bees from those remaining in the hive.

The number of bees sampled at each time point varied with the health of the colony. We attempted to collect at least 60 bees per sample interval, until the end, when too few bees remained to take even ten bees, which is the minimum sample size used for proteomics analysis. We also recorded forager flight activity and forager losses using a bi-directional digital bee-counter mounted at the entrance of the observation colony.

### Inoculation Experiments

We are working on isolating the IIV that infects CCD bees for use in inoculations to perform Koch's Postulates. For our preliminary experiments, and because of the high degree of similarity between the CCD-related IIV and IIV-6, based on the MSP data, we elected to conduct inoculation trials using IIV-6 and *Nosema ceranae* to observe how these two pathogens may interact.

Bees were obtained from non-CCD colonies with no detectable levels of *Nosema*, as confirmed by PCR, from the MSU apiary. *Nosema ceranae* was obtained from local colonies known to be infected by the microsporidian. The New Zealand strain of IIV-6 was obtained from Dr. James Kalmakoff, reared in *Galleria mellonella* larvae, and purified on sucrose density gradients as described previously [55].

Following emergence from brood frames in an incubator, 1–3 day old bees were placed into sterile cardboard cups in a plant growth chamber with controlled temperature (28°C, relative humidity, and light). Using a 10 µl pipette, each bee was inoculated by feeding it a total of 2 µl in sugar water containing one of four treatments. Only bees that ingested the entire inoculum were used.

The following treatments were given: 1) **Controls** = Sugar Water/PBS 1∶1, 2) ***Nosema ceranae*** – 2 µl containing 50,000 spores, 3) **Virus** – 2 µl of 0.25 mg/ml IIV-6 suspension in PBS/Sugar Water 1∶1 (0.25 ug Virus), and 4) ***N. ceranae ***+ **Virus** - 2 µl containing 50,000 spores+0.25 µg of virus in PBS/Sugar Water 1∶1.

Thirty bees were inoculated in each group and the experiment was repeated four separate times for a total of 120 bees in each group. Bees that perished 24 hours after the inoculation were not included in the statistical analysis. Bees were then monitored daily for a period of 14 days. Dead bees were removed immediately upon discovery and frozen at −80°C.

Dead bees from the inoculation experiments were analyzed by PCR and proteomics to detect and confirm infections by *N. ceranae* and virus. We used Kaplan-Meier Curve analyses and Log-Rank Test statistics to determine the significance of the mortality results.

### MSP protocols for double-blinded samples

Bee samples were homogenized in 100 mM of ammonium acetate buffer using a tissue homogenizer. The supernatant was filtered to remove large particulates, followed by ultrafiltration at 300 kDa. All filtered bee samples were lysed using an ultra-sonication probe at settings of 20 seconds pulse-ON, 5 seconds pulse-OFF, and 25 percent amplitude for 5 minutes duration. To verify cells were appropriately disrupted, a small portion of lysates was subjected to 1-D gel analysis. The lysates were centrifuged at 14,100× g for 30 minutes to remove all cellular debris. Supernatant was then added to a Microcon YM-3 filter unit (Millipore; USA) and centrifuged at 14,100× g for 30 minutes. Effluent was discarded and the filtrate were denatured by adding 8 M urea and 3µg/µl dithiothreitol (DTT) and incubated for two hours in an orbital shaker set to 50°C and 60 rpm.

A 10 µL volume of 100 percent acetonitrile (ACN) was added to tubes and allowed to sit at room temperature for 5 minutes. Tubes were washed using 100 mM ABC solution and then spun down at 14,100× *g* for 30–40 minutes. The isolated proteins were then digested with 5 µl trypsin at a solution of 1 µg/µl (Promega, USA) in 240 µl of ABC solution + 5 µl ACN. Digestion was performed overnight at 37°C in an orbital shaker set to 60 rpm. Sixty microliters of 5 percent ACN/0.5 percent formic acid (FA) was added to each filter and vortex mixed gently for 10 minutes. Tubes were centrifuged for 20–30 minutes at 14,100× *g*. An additional 60 µl 5 percent ACN/0.5 percent FA mixture was added to filter and spun. Effluent was then analyzed using the LC-MS/MS technique.

A protein database was constructed in a FASTA format using the annotated bacterial and viral proteome sequences derived from all fully sequenced chromosomes of bacteria and viruses, including their sequenced plasmids (as of September 2008) [16], [17]. A PERL program (http://www.activestate.com/Products/ActivePerl) was written to automatically download these sequences from the National Institutes of Health National Center for Biotechnology (NCBI) site (http://www.ncbi.nlm.nih.gov).

Each database protein sequence was supplemented with information about a source organism and a genomic position of the respective ORF embedded into a header line. The database of bacterial proteomes was constructed by translating putative protein-coding genes and consists of tens of millions of amino acid sequences of potential tryptic peptides obtained by the *in silico* digestion of all proteins (assuming up to two missed cleavages).

The experimental MS/MS spectral data of bacterial peptides were searched using the SEQUEST® (Thermofisher Scientific, USA) algorithm against a constructed proteome database of microorganisms. SEQUEST thresholds for searching the product ion mass spectra of peptides were Xcorr, deltaCn, Sp, RSp and deltaMpep. These parameters provided a uniform matching score of all candidate peptides [17], [18]. The generated outfiles of these candidate peptides were then validated using peptide prophet algorithm.

This validating and verification approach uses an expectation-maximization algorithm as described by the Keller *et al.* [18], the creators of PeptideProphet. The algorithm calculates a statistical score that reflects the confidence of the match to each peptide identified. Peptides identified are eliminated if they are below a selected threshold. In our case, the threshold was set at 95 percent. Peptides that were identified with less than 95 percent confidence were removed from the final data set.

Peptide sequences with probability score of 95 percent and higher were retained in the dataset and used to generate a binary matrix of sequence-to-microbe assignments. The binary matrix assignment was populated by matching the peptides with corresponding proteins in the database and assigned a score of 0 (no-match) or 1 (match). The column in the binary matrix represented the proteome of a given microbe and each row represented a tryptic peptide sequence from the LC-MS/MS analysis.

Bee samples were identified with the virus/bacterium/fungi proteome based on the number of unique peptides that remained after removal of degenerate peptides from the binary matrix. This approach was successfully used for the double-blind characterization of non-genome-sequenced bacteria by mass-spectrometry-based protoemics [19].

Proteomics identified peptides described from a variety of bee viruses, as well nine species of *Nosema: N. apis*, *N. bombycis*, *N. locustae* (now known as *Antonospora locustae*), *N. tricoplusiae*, *N. BZ-2006B*, *N. BZ-2006d*, *N. granulosis*, *N. empoascae*, *N. putellae*, plus a collection of unspecified *Nosema* peptides. At the time that our proteomics analyses were conducted, the *N. ceranae* genome sequence was not available and only one *N. ceranae* sequence was available in the database.

It is almost certain that the diversity of *Nosema* represented in the proteomics results was not attributable to multiple infections by all the species identified. Rather, the taxonomic diversity in the data reflects historical precedent in the *Nosema* research that added different proteins to the genomic and proteomic libraries. Assuming that the *Nosema* proteome described in our data indicated one or at most a few species, we entered total peptide counts for each species into a hierarchical cluster analysis using average Chi-squared distance between species. The analysis produced two primary groupings of *Nosema* peptides: Group 1 which contained *N. apis*, *N. bombycis*, and *N. locustae*; and Group 2 which contained all of the remaining *Nosema* species.

We performed forward, stepwise discriminant analysis on square-root or log transformed pathogen counts for *Nosema* species and for all of the bee virus species. Counts were calculated by weighting each pathogen occurrence by the total number of its peptides that were detected. The specific transform performed on each variable was the one that best normalized the distribution of individual variables.

Use of peptide counts as a weighting factor stems from the observation that as total pathogen titer in a sample increases, the number of different peptides that can be identified by proteomics increases in a predictable manner [18]. Thus, the number of peptides observed for each pathogen served as a relative measure of its abundance in the sample.

Four colony groups were discriminated: strong, failing, collapsed, and the Montana reference group. Selection method for variable entry was largest univariate *F*-value; *F* to enter was set at 2.0. Equal probability of group membership was assumed.

The analysis was completed after two steps including only IIV-6 and DFW as significant discriminating variables (Final Wilks' lambda = 0.679; *F* = 2.881; df = 2, 54; *P* = 0.031; Table 2). None of the *Nosema* groups were selected for the discriminant functions, but *Nosema* 1 was strongly correlated with IIV; the pooled within groups correlation matrix from which the discriminant functions were extracted showed the highest among groups correlation was between IIV and *Nosema* 1 (*r* = 0.89). Because IIV and *Nosema* 1 conveyed the same discriminating information, only one was included.
